# Supplementary material for: Preliminary Study on the Protective Effects and Molecular Mechanism of Procyanidins against PFOS-Induced Glucose-Stimulated Insulin Secretion Impairment in INS-1 Cells
Source: Toxics. 2023 Feb 14;11(2):174. doi: 10.3390/toxics11020174 (PMC9966006; doi:10.3390/toxics11020174)
Supplement: Supplementary file 1 [file toxics-11-00174-s001.zip › toxics-2163418-supplementary.pdf]

Supplemental Files

Table S1. Main experimental materials, reagents and instruments.

Materials	Source Information
INS-1 cells	Cell Resource Center of Shanghai Institutes of Biological Sciences, Chinese Academy of Sciences, Shanghai, China
RPMI-1640 basal medium<br>Fetal bovine serum (FBS)<br>Penicillin–streptomycin solution	Biological Industries, Beth Haemek, Israel
Trypsin<br>D-glucose<br>Sodium pyruvate<br>β-mercaptoethanol	Beijing Solarbio Science & Technology Co., Ltd., Beijing, China
PFOS (CASRN: 1763-23-1, lot: 33607, purity: ≥99%)	Sigma-Aldrich, St. Louis, MO, USA
Procyanidins (CASRN: 4852-22-6, lot: S0208A, purity: ≥95%)	Melone Pharmaceutical Co., Ltd., Dalian, China
0.22 μm syringe filter	Millipore Corp, Billerica, MA, USA
Whole-cell lysis buffer<br>Cell Counting Kit-8<br>BCA Protein Quantitation Kit<br>ROS Detection Kit	NanJing KeyGen Biotech Co., Ltd., Nanjing, China
Rat Insulin ELISA Kit	RayBiotech, Norcross, GA, USA
RNAsimple Total RNA Kit<br>FastKing gDNA Dispelling RT SuperMix Kit<br>Real Universal Color Premix (SYBR Green) Kit	TIANGEN Biotech (Beijing) Co., Ltd., Beijing, China
Horseradish peroxidase-labeled IgG<br>Rabbit anti-β-actin polyclonal antibody	Beijing Zhongshan Jinqiao Biotechnology Co., Ltd., Beijing, China
Rabbit anti-insulin monoclonal antibody	Abcam, Cambridge, MA, USA
Primers	Sangon Biotech (Shanghai) Co., Ltd., Shanghai, China
CO_2 incubator (HERAcell 240i)<br>Multiskan Spectrum (Multiskan GO)<br>Ultramicro ultraviolet spectrophotometer (NanoDrop One)	Thermo Fisher Scientific, Waltham, MA, USA
Electrophoresis system (Mini-PROTEAN Tetra Cell)<br>Real-time PCR detection system (CFX Connect Thermal Cycler)<br>Enhanced chemiluminescence detection system	Bio-Rad, Hercules, CA, USA
Multimode plate reader (VICTOR Nivo)	PerkinElmer, Norwalk, CT, USA

Table S2. Primer's sequences.

Gene	GenBank No.	Forward primers (5′–3′)	Reverse primers (5′–3′)	Amplification product size
β-actin	NM_031144.3	GACTACCTCATG AAGATCCTGACC	TCTCTTTAATGT CACGCACGATT	85bp
Glut2	NM_012879.2	CGGCTGTCTCTGT GCTGCTTGT	GCCGTCATGCT CACATAACTCA	151bp
Gck	NM_012565.2	GCTTTTGAGACC CGTTTCGT	CGCACAATGTC GCAGTCG	119bp
Insulin	NM_019130.2	TCTTCTACACAC CCATGTCCC	GGTGCAGCACT GATCCAC	149bp

Methods

Molecular docking simulation

The protein structures were downloaded from the UniProt database and RCSB PDB database (http://www.rcsb.org/pdb), and the SDF format of PFOS was downloaded from PubChem (https://pubchem.ncbi.nlm.nih.gov/) and converted into Mol2 format in Open Babel. PyMOL software was used to remove water molecules and small molecule ligands in protein, and the AutoDockTools1.5.6 was used for molecular docking of PFOS and protein. The binding energy was calculated by AutoGrid. In order to improve the accuracy of calculation, the parameter "number of GA runs" was set to 50, the output method was set to Lamarckian genetic algorithm, and the rest of the parameters were set as default values. Subsequently, the small molecules in the optimal conformation after docking were compared with the small molecules of the original ligand. When the root mean square deviation (RMSD) were less than 2Å, it was considered that the docking method was feasible and the results were credible.

Results

Molecular docking results of PFOS with the proteins encoded by *GCK*, *GLUT2*, and *INSR* (insulin receptor) genes

The results of molecular docking showed that PFOS could bind to the protein molecule encoded by GCK (*Homo sapiens*) and form hydrogen bonds with Arg-35, Agr-36, and Lys-32 near the active site when the binding energy (less than −1.2 kcal/mol) and RMSD value (less than 2Å) were considered at the same time (Table S3 and Figure S1).

Table S3. Molecular docking results of PFOS with proteins encoded by *GCK*, *GLUT2*, and *INSR* genes

Gene Name	Protein ID (UniProt/RCSB PDB)	Binding Energy (kcal/mol)	RMSD (Å)
Gck (Rattus norvegicus)	AF-P17712-F1 (no ligand) / —	-1.66	—
Gck (Mus musculus)	AF-P52792-F1 (no ligand) / —	-2.12	—
GCK (Homo Sapiens)	4dch	-3	1.401
Glut2 (Rattus norvegicus)	AF-P12336-F1 (no ligand) / —	-2.02	—
Glut2 (Mus musculus)	AF-P14246-F1 (no ligand) / —	-2.76	—
GLUT2 (Homo Sapiens)	AF-P11168-F1 (no ligand) / —	—	—
Insr (Rattus norvegicus)	— / 4xst	— / -2.57	— / 2.408
Insr (Mus musculus)	AF-P15208-F1 (no ligand) /—	—	—
INSR (Homo Sapiens)	— / 1i44, 5hhw	— / -2.46, -3.24	— / 2.313, 3.001

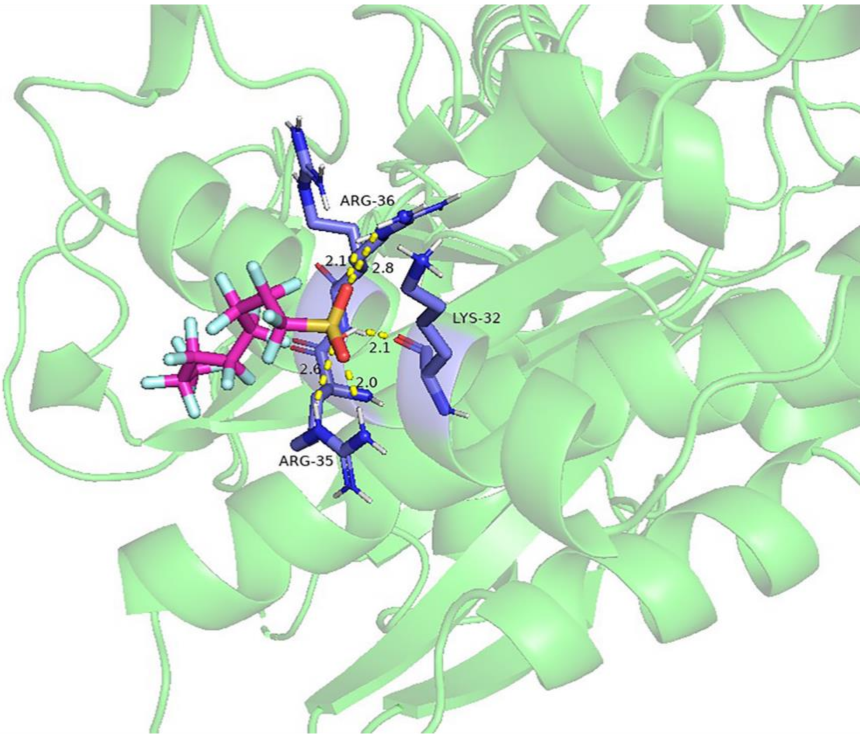

Figure S1. Molecular docking results of PFOS with the protein encoded by *GCK* (*Homo Sapiens*). PFOS is shown as sticks (C: magenta, F: cyan, S: yellow, O: red, H: white). The protein encoded by *GCK* is shown as a green helix. The amino acid residues of the protein are shown as marine-blue sticks. The hydrogen bonds are indicated by yellow dotted lines.
